# No reliable gray matter alterations in idiopathic dystonia

**DOI:** 10.3389/fneur.2025.1510115

**Published:** 2025-03-03

**Authors:** Zhen-Yu Wang, Fei Chen, Hai-Hua Sun, Hua-Liang Li, Jian-Bin Hu, Zhen-Yu Dai, Shu Wang

**Affiliations:** ^1^Department of Radiology, Affiliated Hospital 6 of Nantong University, Yancheng Third People’s Hospital, Yancheng, China; ^2^Department of Neurology, Affiliated Hospital 6 of Nantong University, Yancheng Third People’s Hospital, Yancheng, China

**Keywords:** idiopathic dystonia, voxel-based morphometry, gray matter, coordinate-based meta-analysis, seed-based d mapping

## Abstract

**Background:**

The structural brain abnormalities associated with idiopathic dystonia (ID) remain inadequately understood. Previous voxel-based morphometry (VBM) studies examining whole-brain gray matter (GM) volume alterations in patients with ID have reported inconsistent and occasionally contradictory findings.

**Methods:**

We performed a coordinate-based meta-analysis (CBMA) using the latest seed-based d mapping with permutation of subject images (SDM-PSI) technique to identify consistent GM alterations in patients with ID at the whole-brain level. Additionally, meta-regression analyses were conducted to explore the potential moderating effects of age, gender, and disease duration on GM volume.

**Results:**

The CBMA incorporated 27 VBM studies, comprising 32 datasets with a total of 840 patients with ID and 834 healthy controls. Our analysis did not identify consistent or reliable GM alterations in patients with ID. The robustness of these findings was confirmed through a jackknife sensitivity analysis. Meta-regression analyses revealed that disease duration significantly influenced GM volume in the right insula.

**Conclusion:**

Based on the best practice guidelines for CBMA, we utilized the most recent SDM-PSI algorithm to perform a new CBMA that included a larger group of individuals with ID. However, in contrast to previous CBMAs, we did not observe any consistent alterations in GM in ID. The findings suggest that using GM volume assessed by VBM as an imaging marker for ID may not be reliable. This could be attributed to ID being a functional disorder, or the inconsistency in GM alterations may be influenced by demographic and clinical variations, differences in imaging protocols and analysis methods, or small sample sizes. It is imperative to control for subject characteristics, employ standardized VBM methodologies, and enhance sample sizes in future research.

## 1 Introduction

Dystonia is the third most prevalent movement disorder worldwide, characterized by abnormal postures or movements in specific regions of the body resulting from persistent muscle contractions (1, 2). The global incidence of dystonia is approximately 30.85 per 100,000 individuals, significantly affecting patients’ quality of life and imposing considerable social and economic burdens (3). Idiopathic dystonia (ID), a common subtype of dystonia, occurs independently of other neurological or genetic conditions (4). While dysfunction in the basal ganglia or cerebello-thalamo-cortical circuits is widely regarded as the primary cause of ID (5, 6), its precise neuropathological and physiological mechanisms remain incompletely understood (7).

Voxel-based morphometry (VBM) facilitates automated comparisons of gray matter (GM) volume or density in T1-weighted magnetic resonance imaging (MRI) scans at the whole-brain level between different groups (8). Unlike the traditional region-of-interest (ROI) approach, VBM offers greater objectivity and efficiency (8, 9). Previous studies have investigated GM alterations in ID using VBM, but the results have been inconsistent and occasionally contradictory. For instance, Chirumamilla et al. and Bianchi et al. reported increased GM volume in the anterior cingulate cortex in patients with ID (10, 11), whereas Piccinin et al. found a decrease in GM in the same region (12). Additionally, some studies have reported no significant GM abnormalities in ID (13–17). Therefore, further research is required to identify consistent and reliable patterns of GM alterations, which could provide deeper insights into the pathophysiological mechanisms underlying ID.

Coordinate-based meta-analysis (CBMA) enables the integration of multiple neuroimaging studies, facilitating the derivation of more generalizable and robust findings (18, 19). To date, five CBMAs have summarized whole-brain VBM studies on ID and its subtypes, yet inconsistencies in their results have been observed (20–24). This discrepancy may be attributed to the use of different CBMA algorithms. Zheng et al. and Wu et al. employed activation likelihood estimation (ALE) techniques (21, 24), whereas Huang et al. and Zhang et al. utilized anisotropic effect size-based signed differential mapping (AES-SDM) techniques (20, 22, 23). In contrast to ALE, AES-SDM can incorporate both significant and non-significant results along with their effect sizes but adopts a less stringent statistical approach (25, 26). Disparities in literature search strategies, time frames, and the focus on specific ID subtypes may have further contributed to these inconsistencies. A comprehensive summary of the information from these five CBMA studies is provided in Table 1.

**Table 1:** The specific information of previous CBMA of GM abnormalities in patients with ID.

Study	Dystonia subtype	Sample (number)	CBMA tool	Corrected	Statistical threshold	Result (GM alterations)
Zheng et al., 2012 (24)	FPD	Patients (199)	ALE, 2.0.4	FDR	*p* < 0.05	Increase: caudate, postcentral cortex, and primary motor cortex
		Controls (247)				Decrease: thalamus and putamen
Wu et al., 2022 (21)	IID	Patients (347)	ALE, 3.0.2	FWE	*p* < 0.05	Increase: left medial and lateral globus pallidus
		Controls (361)				
Huang et al., 2022 (23)	ID	Patients (701)	AES-SDM	Uncorrected	*p* < 0.005	Increase: bilateral precentral and postcentral gyri, bilateral putamen and pallidum, right insula, and left supramarginal gyrus
		Controls (712)				Decrease: bilateral temporal poles, bilateral supplementary motor areas, right angular gyrus, inferior parietal gyrus and precuneus, left insula, and inferior frontal gyrus
Huang et al., 2022 (22)	iCD	Patients (152)	AES-SDM, 5.15	Uncorrected	*p* < 0.005	Increase: bilateral thalamus and caudate nuclei, right precentral gyrus, right supplementary motor area, right paracentral lobule and dorsolateral superior frontal gyrus
		Controls (188)				Decrease: left cerebellum, left middle temporal gyrus and dorsolateral superior frontal gyrus, right angular gyrus and inferior parietal gyrus
Zhang et al., 2022 (20)	iBSP	Patients (129)	AES-SDM	Uncorrected	*p* < 0.005	Increase: bilateral precentral and postcentral gyri, right supplementary motor area, and bilateral paracentral lobules
		Controls (144)				Decrease: right superior and inferior parietal gyri, left inferior parietal gyrus, left inferior temporal gyrus, left fusiform gyrus, and parahippocampal gyrus
Our CBMA	ID	Patients (840)	SDM-PSI, 6.23	TFCE-FWE	*p* < 0.05	Did not find any significant brain regions
		Controls (834)				

CBMA, coordinate-based meta-analysis; GM, gray matter; ID, idiopathic dystonia; FPD, primary focal dystonia; IID, isolated idiopathic dystonia; iCD, idiopathic cervical dystonia; iBSP, idiopathic blepharospasm; ALE, activation likelihood estimation; AES-SDM, anisotropic effect size-based signed differential mapping; SDM-PSI, seed-based d mapping with permutation of subject images; FDR, false discovery rate; FWE, family-wise error; TFCE-FWE, threshold-free cluster enhancement-based family-wise error.

In line with the latest guidelines and recommendations, several aspects of the previous five CBMAs require improvement (27–29). First, the studies by Gellea et al. and Pantano et al. should be excluded, as they employed ROI techniques rather than whole-brain approaches in their VBM analyses (30, 31). Additionally, the inclusion of studies that employed small-volume correction (SVC) should be reassessed (32–36), as this method may exaggerate results in specific brain regions and introduce bias in region selection (27). Furthermore, Zheng et al. applied an older version of ALE with false discovery rate (FDR) correction (24), which has been demonstrated to be potentially suboptimal for CBMA (37). Moreover, the inclusion of ALE analyses with a limited number of studies (9 and 14 studies, respectively) (21, 24), while a minimum of 17 studies is recommended for ALE analysis to ensure adequate statistical power (38). These factors likely compromised the robustness of the CBMA results. Notably, the number of VBM studies focusing on ID has increased in recent years. Thus, an updated CBMA of existing VBM studies on ID is crucial for identifying consistent and robust GM alterations.

The latest version of CBMA, seed-based d mapping with permutation of subject images (SDM-PSI), applies threshold-free cluster enhancement (TFCE)-based family-wise error (FWE) correction to control for multiple comparisons (39). This method is advantageous because it simultaneously addresses both FWE and cluster-level significance without requiring a predefined cluster size threshold, thus preserving statistical sensitivity while reducing the likelihood of false positive findings. It has demonstrated effectiveness in a range of neurological and psychiatric disorders, including schizophrenia and Parkinson’s disease, and has exhibited high statistical efficacy (40, 41). Therefore, we conducted an updated CBMA to identify consistent and robust GM alterations in ID, employing SDM-PSI in line with current guidelines and recommendations (27–29). Additionally, meta-regression analyses were performed to examine the potential influence of participant demographic and clinical characteristics on GM alterations.

## 2 Methods

### 2.1 Study search and selection

A systematic and comprehensive search was conducted across the PubMed, Web of Science, and Embase databases to identify relevant studies published up to September 21, 2024. The search used the keywords (“dystonia” OR “blepharospasm” OR “writer’s cramp” OR “spasmodic dysphonia” OR “Meige’s syndrome” OR “dystonic disorders”) AND (“voxel*” OR “VBM” OR “morphometry”). Additionally, potential studies were sourced through literature review and scrutiny of references in the retrieved articles. Studies that met the following criteria were included: (1) original peer-reviewed English-language articles; (2) applying VBM analysis to compare GM differences between patients with ID and healthy controls (HC) at the whole-brain level (ROI analysis or SVC analysis were not included); and (3) findings reported in stereotactic three-dimensional coordinates (x, y, z). In cases of overlapping subjects among studies, the study with the larger sample size was prioritized for inclusion. Studies were excluded if the required data could not be obtained despite efforts to contact corresponding authors.

### 2.2 Data extraction

Data were collected on participant characteristics (ID subtype, sample size, age, gender, handedness, and disease duration), imaging details (e.g., MRI scanner, magnetic field strength, sequence, voxel size, head coil channels, processing software, modulation, Gaussian kernel, covariates, and statistical threshold), and peak coordinates and *t*-values of regions showing significant GM alterations compared to HC. In cases where z- or *p*-values were provided, the online SDM tool was utilized to convert them to *t*-values. Two researchers independently reviewed and extracted data from the studies, resolving discrepancies through discussion. In instances of unresolved differences, a third author was consulted for consensus.

### 2.3 Quality assessment

The quality of the studies was assessed using a 10-point checklist (42), with primary evaluation criteria including sample size, demographic information, key clinical variables, methodological thoroughness, result presentation, and study limitations. Each criterion received a score of 0, 0.5, or 1, indicating non-compliance, partial compliance, or full compliance with the specified criteria. Two authors independently assessed and scored each study, resolving any discrepancies through discussion. The checklist details are available in Supplementary Table S1.

### 2.4 CBMA of included VBM studies

The SDM-PSI software version 6.23^1^ was used to perform the meta-analysis. A detailed description of the SDM-PSI approach is available in previous publications (39, 43). The main steps are briefly outlined as follows: Initially, a document was created to compile the peak coordinates, effect sizes, and demographic/clinical details (e.g., sample size, age, gender, disease duration, etc.) from all original studies. Second, the upper and lower bounds of possible effect size images were calculated within a GM mask. Third, effect size analysis was conducted using the multiple imputation algorithm MetaNSUE. Fourth, Rubin’s rule was employed to voxel-wise combine meta-analysis images from various input datasets. Finally, subject images were reconstructed for permutation tests, with multiple comparisons corrected using TFCE-based FWE correction (cluster-level *p* < 0.05, voxel extent ≥10).

### 2.5 Sensitivity, heterogeneity and publication bias analyses

A jackknife sensitivity analysis was conducted on the datasets to evaluate the impact of individual studies on the overall outcomes. By systematically excluding one dataset at a time and conducting iterative analyses, brain regions that consistently showed significance were deemed highly reproducible (44). Heterogeneity across the studies was assessed using the I^2^ statistic, with an I^2^ value less than 50% indicating low heterogeneity (45). Additionally, Egger’s test was employed to examine potential publication bias.

### 2.6 Meta-regression analyses

Linear meta-regression analyses were conducted to investigate the impact of diverse demographic and clinical factors, with particular attention to age, gender, and disease duration. A statistical significance threshold of *p* < 0.05 (TFCE-based FWE correction) and a voxel extent ≥10 was applied.

## 3 Results

### 3.1 Information on included studies and participants

**Figure 1:**
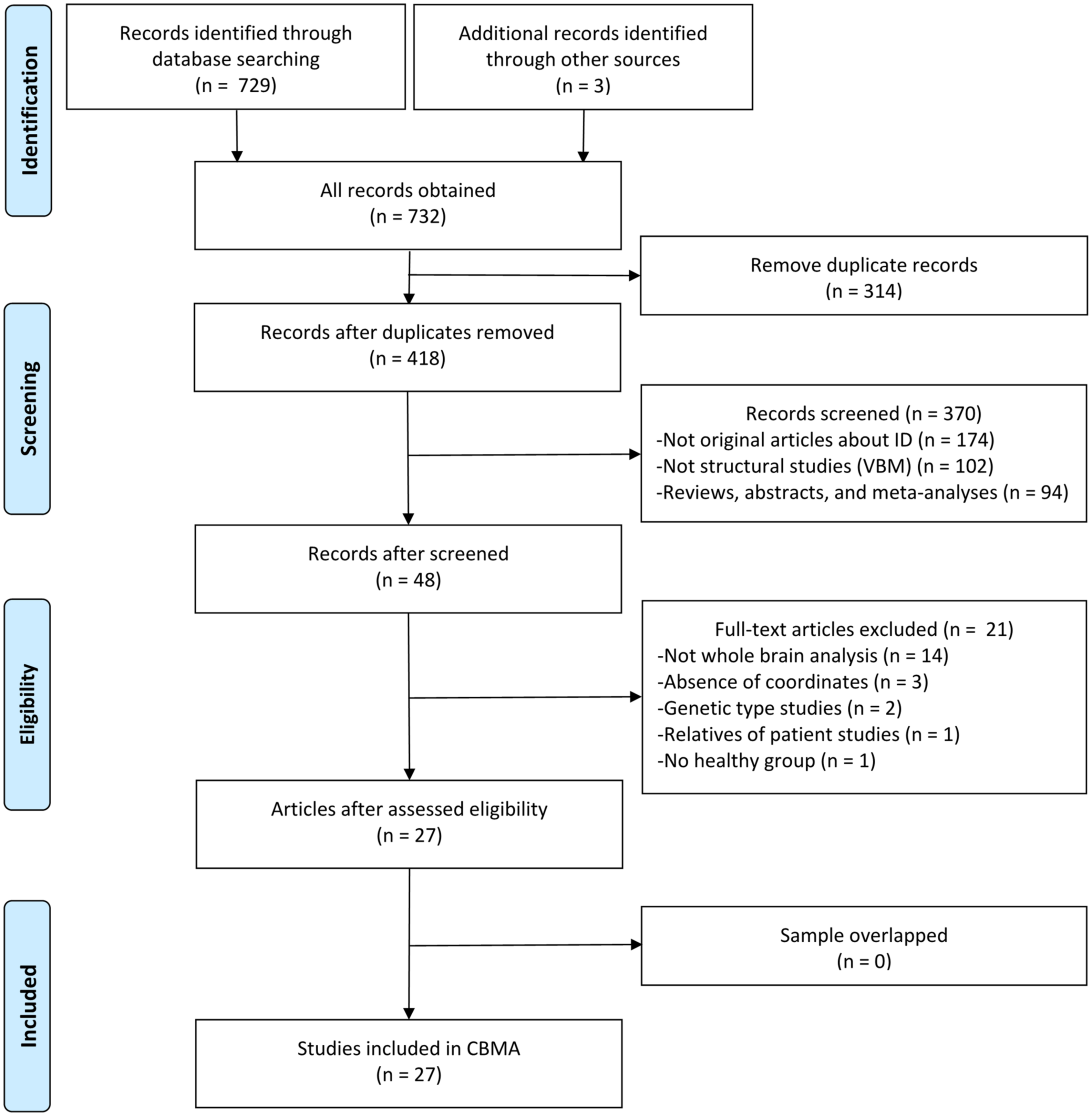
Literature selection flowchart in meta-analysis. ID, idiopathic dystonia; VBM, voxel-based morphometry; CBMA, coordinate-based meta-analysis.

A total of 730 results were identified through the literature search, leading to the inclusion of 27 original VBM studies in this CBMA (10–17, 21, 46–63). The comprehensive literature screening process is outlined in Figure 1. Of the incorporated studies, 21 reported increases or decreases in GM volume in specific brain regions, while 6 reported no significant alterations in patients with ID. These studies comprised 32 datasets, encompassing a total of 840 patients with ID and 834 HC subjects. Sample sizes across these datasets ranged from 7 to 73 for the patient groups (mean: 26.25) and from 7 to 83 for the HC groups (mean: 26.06). Reported demographic and clinical characteristics included gender (62.6% female in the patient groups and 58.3% in the HC groups across 32 datasets), age (mean: 58.02 years for patients, 52.42 years for HC across 31 datasets), and disease duration (mean: 9.15 years across 28 datasets). The demographic and clinical data for each study are presented in Table 2. Furthermore, Table 3 outlines the imaging protocols and data processing methods. Each study underwent a quality assessment, with detailed scores provided in Supplementary Table S2.

**Table 2:** Demographic and clinical characteristics of VBM studies in the meta-analysis.

Study	Type	Number (Female)	Handedness (R/L)	Age (standard deviation), years	Duration (standard deviation), years	Quality score
Etgen et al., 2006 (46)	BSP	16 (12)	R	67.4 (4.3)	6.5 (4.9)	8.5
Delmaire et al., 2007 (47)	WC	30 (21)	R	49.7 (12.9)	7 (6.5)	8.5
Egger et al., 2007 (48)	PD	31 (11)	R	43.85 (NA)	9.47 (NA)	9.5
Obermann et al., 2007 (49)	CD	9 (7)	R	57.6 (7.2)	10 (6.8)	9.5
	BSP	11 (7)	R	52.6 (10.6)	5.5 (4.3)	
Martino et al., 2011 (50)	BSP	25 (17)	R	64.9 (7.8)	7.8 (6.2)	9.5
Suzuki et al., 2011 (51)	BSP	32 (22)	NA	55.0 (6.5)	5.5 (4.6)	9
Horovitz et al., 2012 (52)	BSP	14 (14)	R (13)/L (1)	59.9 (6.1)	3.21 (5.74)	9
Simonyan et al., 2012 (53)	SD	40 (25)	NA	56.9 (10.06)	14.4 (NA)	10
Prell et al., 2013 (54)	CD	24 (18)	NA	52 (NA)	13.9 (10.4)	10
Yang et al., 2013 (17)	BSP	18 (14)	R	55.54 (8.42)	3.83 (3.93)	10
Cerasa et al., 2014 (55)	DT	12 (6)	NA	62.9 (15)	10.9 (8.9)	9
Ramdhani et al., 2014 (56)	TSD	24 (14)	R	53.75 (NA)	13.35 (10.63)	10
	NTSD	21 (16)	R	58.47 (NA)	9.92 (7.57)	
Delnooz et al., 2015 (57)	CD	23 (14)	R (21)/L (2)	57.3 (9.8)	12.7 (7.2)	9
Piccinin et al., 2015 (12)	CCD	27 (18)	NA	54.18 (4.7)	11.37 (6.87)	9
Zeuner et al., 2015 (58)	WC	22 (13)	NA	50.7 (12.2)	14.2 (7.6)	10
Waugh et al., 2016 (16)	CD	17 (11)	R (16)/L (1)	52 (2.3)	NA	9.5
	SD	7 (6)	R	53 (2.9)	NA	
Burciu et al., 2017 (15)	CD	16 (11)	R (15)/L (1)	57.6 (11.5)	5.8 (4)	8.5
Kirke et al., 2017 (59)	SD	40 (34)	R	57.2 (NA)	13.1 (NA)	9.5
Mantel et al., 2018 (60)	WC	26 (11)	NA	46.8 (13.7)	13.2 (10.8)	10
Bianchi et al., 2019 (11)	TSFD	16 (8)	R	43.5 (10.8)	12.7 (10.6)	9.5
Chirumamilla et al., 2019 (10)	BSP	13 (8)	NA	65 (6)	NA	9
Gracien et al., 2019 (14)	CD	17 (9)	NA	51 (8.9)	NA	9
Mantel et al., 2019 (61)	ED	24 (3)	NA	43.5 (11.2)	7.2 (6.7)	9
Liu et al., 2020 (62)	MS	46 (35)	NA	57 (8.86)	4.57 (2.23)	10
Tomic et al., 2021 (63)	TSD	36 (22)	R	53.9 (12.2)	9 (7.9)	10
	NTSD	61 (42)	R	57 (11.6)	7.9 (5.9)	
Wu et al., 2022 (21)	IID	73 (36)	R	43.04 (18.23)	6.23 (6.78)	10
Yang et al., 2024 (13)	AOID with anxiety	35 (23)	NA	NA	NA	10
	AOID	34 (18)	NA	NA	NA	

VBM, voxel-based morphometry; R, right; L, left; BSP, blepharospasm; WC, writer’s cramp; PD, primary dystonia; CD, cervical dystonia; SD, spasmodic dysphonia; DT, dystonic tremor; TSD, task-specific dystonia; NTSD, non–task-specific dystonia; CCD, craniocervical dystonia; TSFD, task-specific focal dystonia; ED, embouchure dystonia; MS, Meige syndrome; IID, isolated idiopathic dystonia; AOID, adult-onset isolated dystonia; NA, not available.

**Table 3:** Imaging and data processing characteristics of the VBM studies in the meta-analysis.

Study	MRI scanner	Field strength (Tesla)	MRI sequence	Voxel size (mm^3^)	Head coil	Software	Modulation	FWHM (mm)	Covariates	Threshold
Etgen et al., 2006 (46)	Siemens	1.5	MPRAGE	1*1*1	2	SPM2	Yes	12	Age	*p* < 0.05, uncorrected
Delmaire et al., 2007 (47)	GE	1.5	SPGR	1*1*1.5	NA	SPM2	Yes	12	Total GM, age	*p* < 0.05, corrected
Egger et al., 2007 (48)	Siemens	1.5	FLASH	NA	NA	SPM2	Yes	10	Total GM, global mean voxel value	*p* < 0.05, corrected
Obermann et al., 2007 (49)	Siemens	1.5	MPRAGE	1*1*1	NA	SPM2	Yes	12	NA	*p* < 0.05, corrected
Martino et al., 2011 (50)	GE	3.0	SPGR	NA	NA	SPM8	Yes	8	Age, gender, total GM	*p* < 0.001, uncorrected
Suzuki et al., 2011 (51)	GE	1.5	SPGR	0.94*0.94*1.3	NA	SPM8	No	9	Age	*p* < 0.05, corrected
Horovitz et al., 2012 (52)	GE	3	MPRAGE	NA	8	FSL	Yes	6.9	NA	*p* < 0.01, uncorrected
Simonyan et al., 2012 (53)	GE	3	MPRAGE	NA	8	SPM8	Yes	10	Age, gender, TIV	*p* < 0.01, corrected
Prell et al., 2013 (54)	GE	1.5	SPGR	0.97*0.97*1.5	NA	SPM2	Yes	8	Global mean voxel value	*p* < 0.05, corrected
Yang et al., 2013 (17)	GE	3	SPGR	NA	8	SPM8	Yes	8	TIV	*p* < 0.05, corrected
Cerasa et al., 2014 (55)	GE	3	SPGR	NA	8	SPM8	Yes	8	Age, TIV	*p* < 0.001, uncorrected
Ramdhani et al., 2014 (56)	Phillips	3	MPRAGE	NA	8	SPM8	Yes	8	Age, gender, TIV	*p* < 0.05, corrected
Delnooz et al., 2015 (57)	Siemens	3	MPRAGE	1*1*1	32	SPM8	Yes	10	Age, gender	*p* < 0.05, corrected
Piccinin et al., 2015 (12)	Phillips	3	NA	1*1*1	NA	SPM8	Yes	10	NA	*p* < 0.001, uncorrected
Zeuner et al., 2015 (58)	Phillips	3	Gradient echo	NA	8	SPM8	Yes	12	Age, gender, TIV	*p* < 0.05, corrected
Waugh et al., 2016 (16)	Siemens	3	MPRAGE	NA	NA	FSL	Yes	3	Age	*p* < 0.05, uncorrected
Burciu et al., 2017 (15)	Phillips	3	NA	1*1*1	32	SPM8	No	8	NA	*p* < 0.05, corrected
Kirke et al., 2017 (59)	Phillips	3	MPRAGE	NA	8	SPM8	No	4	Age, gender	*p* < 0.05, corrected
Mantel et al., 2018 (60)	Phillips	3	NA	1*1*1	8	SPM12	Yes	8	Age, gender, TIV	*p* < 0.05, corrected
Bianchi et al., 2019 (11)	Siemens	3	MPRAGE	1*1*1	32	SPM12	No	6	Age, gender, TIV	*p* < 0.01, corrected
Chirumamilla et al., 2019 (10)	Phillips	3	NA	1*1*1	8	SPM8	Yes	8	NA	*p* < 0.001, corrected
Gracien et al., 2019 (14)	Siemens	3	SPGR	1*1*1	8	FSL	No	NA	NA	NA
Mantel et al., 2019 (61)	Phillips	3	MPRAGE	1*1*1	8	SPM12	Yes	10	Age, gender, TIV	*p* < 0.05, corrected
Liu et al., 2020 (62)	GE	3	SPGR	NA	NA	SPM8	No	8	Age, gender, TIV	*p* < 0.001, corrected
Tomic et al., 2021 (63)	Phillips	1.5	TFE	NA	NA	SPM12	Yes	8	Age, BoNT	*p* < 0.05, corrected
Wu et al., 2022 (21)	GE	3	MPRAGE	1*1*1	NA	SPM12	Yes	6	Age, gender, TIV	*p* < 0.005, corrected
Yang et al., 2024 (13)	Siemens	3	NA	1*1*1	NA	NA	Yes	8	Age, gender, TIV	*p* < 0.05, corrected

VBM, voxel-based morphometry; MRI, magnetic resonance imaging; FWHM, full-width-at-half-maximum; BoNT, botulinum toxin; MPRAGE, magnetization prepared rapid gradient echo; SPGR, spoiled gradient recalled echo; FLASH, fast low angle shot; TFE, turbo field echo; NA, not available; SPM, statistical parametric mapping; FSL, FMRIB’s Software Library; GM, gray matter; TIV, total intracranial volume.

### 3.2 CBMA of included VBM studies

No statistically significant and consistent differences in GM were identified between patients with ID and HC subjects across 32 datasets after applying TFCE-based FWE correction (*p* < 0.05, voxel extent ≥10).

### 3.3 Sensitivity, heterogeneity and publication bias analyses

The jackknife sensitivity analysis indicated no consistent alterations in GM between patients with ID and HC subjects across all datasets. Since no significant brain clusters were identified in the CBMA, further analyses of heterogeneity and publication bias were not conducted.

### 3.4 Meta-regression analyses

**Figure 2:**
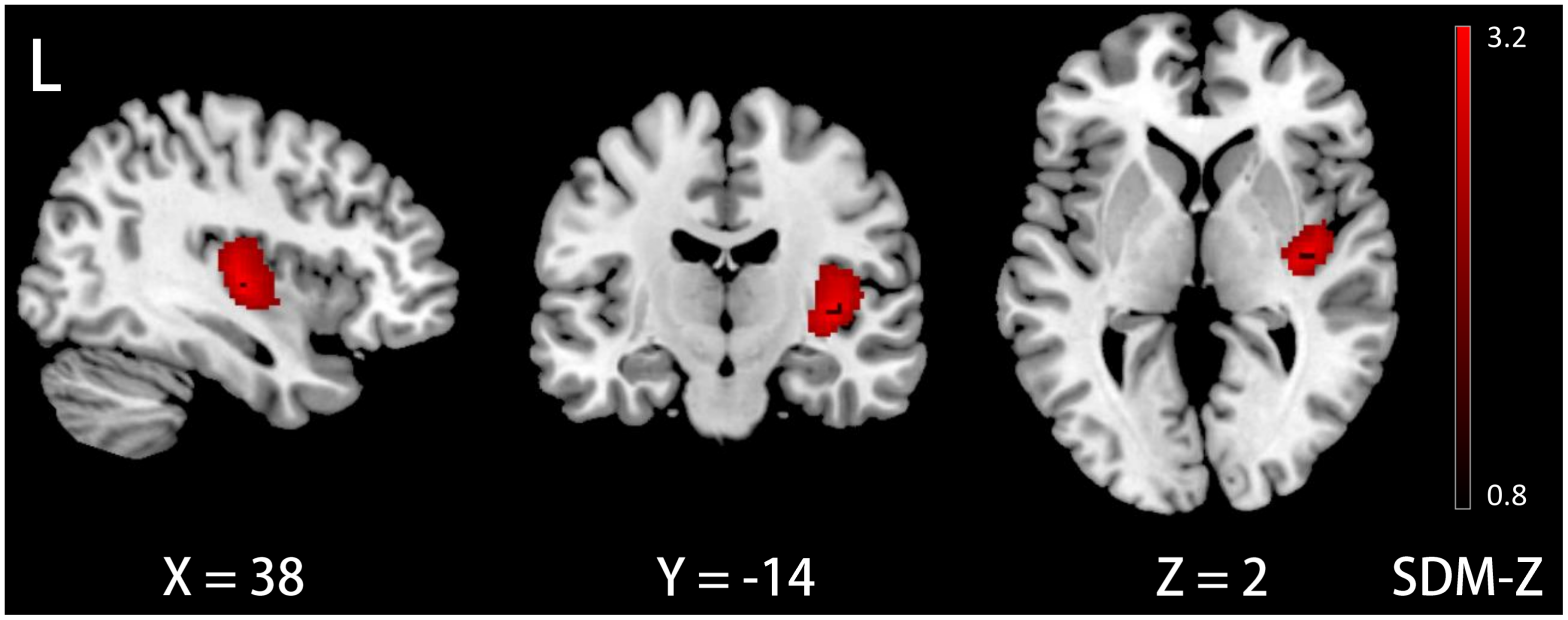
Meta-egression results demonstrating GM volume increases in the right insula with disease duration in ID. GM, gray matter; ID, idiopathic dystonia; L, left.

The meta-regression analysis demonstrated that a longer disease duration, as reported in 28 datasets, was associated with an increase in GM volume in the right insula (Montreal Neurological Institute [MNI] coordinates: x = 38, y = −14, z = 2; Brodmann area 48; SDM-Z = 3.258; voxels = 621; TFCE-based FWE correction, *p* < 0.01, Figure 2). Furthermore, neither age nor gender exhibited associations with GM volume (*p* < 0.05, TFCE-based FWE correction and voxel extent ≥10).

## 4 Discussion

This updated CBMA did not exhibit any consistent GM alterations in patients with ID compared to HC subjects, with jackknife sensitivity analysis confirming the robustness of the findings. Furthermore, meta-regression analysis revealed a significant impact of disease duration on GM in the right insula.

It is noteworthy that our findings were inconsistent with those in previous CBMAs (20–24), which may be attributed to the application of the latest SDM-PSI algorithm alongside more stringent statistical methods, and adherence to contemporary guidelines that excluded studies using ROI and SVC analyses. Additionally, our CBMA included a larger cohort comprising 32 original VBM datasets and 840 participants, thus enhancing the reliability of the results. Consequently, the absence of consistent GM alterations may suggest that GM is not a dependable neuroimaging biomarker for ID.

One potential explanation for the inconsistent GM abnormalities observed in patients with ID may be the absence of true GM alterations within this population. Previous autopsy studies and animal models have demonstrated that ID does not present with structural brain anomalies (64–66). Recent functional MRI studies have revealed significant abnormalities across several brain regions in patients with ID, including the primary motor cortex, supplementary motor area, cerebellum, thalamus, and putamen (67–71). Additionally, several studies have demonstrated a correlation between the severity of clinical symptoms and functional abnormalities in specific brain regions (68, 69, 71). Furthermore, some research suggests that symptomatic treatments, such as botulinum toxin injections or deep brain stimulation, can modulate abnormal brain functions (72–75). Collectively, these findings suggest that ID may be primarily characterized as a functional brain disorder that does not necessarily involve structural abnormalities.

The inconsistency in identifying GM alterations in ID may be attributed to the heterogeneity among participants. As illustrated in Table 2, the 32 datasets include 13 distinct subtypes of ID, highlighting significant diversity in demographic and clinical characteristics. Specific subtypes of ID are often associated with distinct patterns of GM abnormalities (76). For instance, Tomic et al. and Ramdhani et al. observed that, compared to non-task-specific dystonia (blepharospasm and cervical dystonia), task-specific dystonia (laryngeal dystonia and writer’s cramp) was associated with increased GM volume in the primary somatosensory cortex, middle frontal gyrus, temporal lobe, and occipital lobe (56, 63). Moreover, the varied motor symptoms observed in patients are frequently associated with increased GM volume in specific brain regions, providing insights into the potential mechanisms underlying GM alterations in ID (64, 77). As a result, GM alterations in ID present diverse patterns, with the disorder’s inherent heterogeneity likely contributing to the lack of consistent GM findings.

The meta-regression analysis indicated that disease duration has an impact on GM volume in the right insula, a region crucial for emotional perception and sensory processing (78, 79). Individuals with ID frequently experience non-motor symptoms such as chronic pain, anxiety, and depression (80–82). These symptoms have been linked to changes in brain structure in various psychiatric and neurological disorders, often correlating with disease duration (83–85). This suggests that the alterations in GM volume in the insula may reflect an adaptive response to these non-motor symptoms in individuals with ID. Although no significant modulatory effects of age or gender on GM volume were observed, caution should be exercised in interpreting this finding, as it is based on study-level rather than individual-level data. Age and gender are well-established factors that are known to influence brain structure (86–89). Other potential confounding variables, such as handedness (90–92), disease severity (12, 54, 57, 93), age of onset (93), medication history (12, 57, 63), and education level (94), may also contribute to discrepancies in brain structure. Unfortunately, the lack of sufficient data from the original studies limits the ability to systematically investigate these potential variables. Consequently, the demographic and clinical heterogeneity of participants in VBM studies may account for the failure to identify consistent GM alterations.

In addition to participant heterogeneity, the inconsistency in GM alterations may also arise from variations in imaging protocols. As outlined in Table 3, the 27 VBM studies employed a range of MRI scanners (GE, Philips, Siemens), magnetic field strengths (3.0 and 1.5 Tesla), head coil channels (2, 8, 32, and unreported), pulse sequences, and voxel sizes. Focke et al. and Takao et al. found that variations in MRI scanners influenced VBM analysis outcomes (95, 96), and other studies have demonstrated that magnetic field strength also influences results (97–99). Furthermore, differences in head coil channels, pulse sequences, and voxel sizes have been associated with discrepancies in GM measurements (77, 100–102). Moreover, inadequate management of head movement during image acquisition may compromise image quality and result in inaccuracies in GM quantification (103, 104). Notably, only a limited number of original studies rigorously controlled for head movement during MRI scanning or conducted comprehensive visual inspections and manual corrections of images (11–14, 17, 21, 54, 55, 57–59, 61).

The inconsistency in GM alterations may also result from variations in image processing procedures, which are a crucial aspect of VBM analysis. Initially, brain images from all subjects underwent segmentation and registration into standard space, followed by modulation to compensate for image deformation, and a smoothing process (8). Subsequently, statistical analyses were conducted to identify and interpret differences between groups. The VBM studies included in the CBMA employed various software platforms (different versions of SPM and FSL), applying diverse techniques for image segmentation, registration, modulation, and smoothing, which could potentially influence GM measurements (105–111). Correcting for multiple comparisons is crucial in neuroimaging studies to prevent inflated positive results and bias (112). However, seven out of the 32 analyzed datasets employed uncorrected thresholds (12, 16, 46, 50, 52, 55). Moreover, factors such as age, gender, and total intracranial volume serve as important covariates in VBM analysis (113, 114); however, six studies failed to incorporate any covariates in their analyses (10, 12, 14, 15, 49, 52). Therefore, the multitude of methodological decisions involved in each step of VBM analysis may contribute to inconsistencies in results.

Finally, a small sample size can constrain the ability to identify significant findings in neuroimaging studies, potentially reducing the robustness of the results (115). Fusar-Poli et al. observed that VBM studies with smaller sample sizes generally produce fewer significant findings (116). However, the current ID datasets have an average of only 26.25 participants, which reduces the statistical power necessary to detect significant GM differences. Furthermore, the non-normal distribution of the data complicates the situation further, as smaller studies are more prone to producing false positives compared to larger ones (117). This susceptibility compromises the generalizability and stability of the results. Consequently, the limited sample size likely plays a major role in the discrepancies observed in VBM findings, explaining the lack of significant GM abnormalities identified in this CBMA.

Taken together, the inconsistency in observed GM alterations may primarily arise from demographic and clinical heterogeneity, variations in imaging acquisition and analytical methods, and small sample sizes. To enhance the robustness and reproducibility of future studies, the following recommendations are proposed: (1) conduct power analyses prior to VBM studies to determine appropriate sample sizes; encourage multicenter collaborations or data sharing to increase sample sizes; (2) conduct comprehensive assessments of clinical population characteristics to minimize the potential impact of confounding factors on results; and (3) establish and implement standardized imaging acquisition and analytical methodologies, and apply rigorous statistical strategies for analysis.

It is essential to acknowledge several limitations of this study. First, CBMA relies on peak coordinates and corresponding effect sizes reported in VBM studies rather than on original datasets. Some scholars have suggested that image-based meta-analyses or hybrid meta-analyses combining images and coordinates may improve result accuracy (118, 119), although this approach depends on the availability of raw data shared by researchers. Second, the datasets included in this CBMA are incomplete, as unpublished studies, non-English publications, and studies lacking crucial information were excluded, which may have introduced selection bias. Third, the limited number of studies focusing on specific ID subtypes impeded the conduct of subgroup CBMA analyses to explore the variability within the disorder. Fourth, we did not analyze functional MRI studies in ID. Integrating structural and functional neuroimaging could help bridge the gap between the inconsistent GM changes observed and the functional abnormalities reported, which may be beneficial in revealing the neuropathological mechanisms of ID.

## 5 Conclusion

The CBMA of whole-brain VBM studies failed to reveal a consistent and reliable pattern of GM differences between patients with ID and HC subjects. This finding may indicate that GM is not a reliable neuroimaging marker for ID. It is possible that ID may primarily be a functional disorder. Another explanation for CBMA’s inability to detect consistent GM alterations could be the demographic and clinical heterogeneity among participants, along with variations in image acquisition, processing techniques, and small sample sizes. To improve the accuracy of future findings, upcoming VBM studies must rigorously assess potential confounding factors, adhere to standardized protocols for image acquisition and analysis, and aim to increase sample sizes.

## Data Availability

The original contributions presented in the study are included in the article/Supplementary material, further inquiries can be directed to the corresponding authors.

## Footnotes

^1^ www.sdmproject.com
