# Supplementary material for: No reliable gray matter alterations in idiopathic dystonia
Source: Front Neurol. 2025 Mar 3;16:1510115. doi: 10.3389/fneur.2025.1510115 (PMC11911186; doi:10.3389/fneur.2025.1510115)
Supplement: Supplementary file 1 [file Data_Sheet_1.docx]

**Supplementary Material**

**Table S1. 10-point checklist of quality assessment.**

| **Category 1: Participants** |
| --- |
| 1. Patients were evaluated prospectively, specific diagnostic criteria were applied, and demographic data were reported.  2. Comparison participants were evaluated prospectively; psychiatric and medical illnesses were excluded.  3. Important variables (e.g., age, sex, disease duration) were checked either by stratification or statistically.  4. Sample size per group > 7. |
| **Category 2: Methods for image acquisition and analysis** |
| 5. Whole brain analysis was automated without a priori regional selection.  6. Coordinates reported in a standard space.  7. The imaging technique used was clearly described so that it could be reproduced.  8. Measurements were clearly described so that they could be reproduced. |
| **Category 3: Results and conclusions** |
| 9. Statistical parameters for significant and important non-significant differences were provided.  10. Conclusions were consistent with the results obtained and the limitations were discussed. |

Note: The checklist is based on previous meta-analyses.

**Table S2. The detailed quality assessment scores for each study.**

| Studies | 1 | 2 | 3 | 4 | 5 | 6 | 7 | 8 | 9 | 10 | Total |
| --- | --- | --- | --- | --- | --- | --- | --- | --- | --- | --- | --- |
| Etgen et al., 2006 | 1 | 1 | 0.5 | 1 | 1 | 1 | 1 | 0.5 | 1 | 0.5 | 8.5 |
| Delmaire et al., 2007 | 1 | 0.5 | 0.5 | 1 | 1 | 1 | 1 | 1 | 1 | 0.5 | 8.5 |
| Egger et al., 2007 | 1 | 1 | 0.5 | 1 | 1 | 1 | 1 | 1 | 1 | 1 | 9.5 |
| Obermann et al., 2007 | 1 | 1 | 0.5 | 1 | 1 | 1 | 1 | 1 | 1 | 1 | 9.5 |
| Martino et al., 2011 | 1 | 1 | 1 | 1 | 1 | 1 | 0.5 | 1 | 1 | 1 | 9.5 |
| Suzuki et al., 2011 | 1 | 1 | 0.5 | 1 | 1 | 1 | 1 | 1 | 1 | 0.5 | 9 |
| Horovitz et al., 2012 | 1 | 0.5 | 1 | 1 | 1 | 1 | 1 | 1 | 1 | 0.5 | 9 |
| Simonyan et al., 2012 | 1 | 1 | 1 | 1 | 1 | 1 | 1 | 1 | 1 | 1 | 10 |
| Prell et al., 2013 | 1 | 1 | 1 | 1 | 1 | 1 | 1 | 1 | 1 | 1 | 10 |
| Yang et al., 2013 | 1 | 1 | 1 | 1 | 1 | 1 | 1 | 1 | 1 | 1 | 10 |
| Cerasa et al., 2014 | 1 | 1 | 1 | 1 | 1 | 1 | 0 | 1 | 1 | 1 | 9 |
| Ramdhani et al., 2014 | 1 | 1 | 1 | 1 | 1 | 1 | 1 | 1 | 1 | 1 | 10 |
| Delnooz et al., 2015 | 1 | 0.5 | 1 | 1 | 1 | 1 | 1 | 1 | 1 | 0.5 | 9 |
| Piccinin et al., 2015 | 1 | 0.5 | 0.5 | 1 | 1 | 1 | 1 | 1 | 1 | 1 | 9 |
| Zeuner et al., 2015 | 1 | 1 | 1 | 1 | 1 | 1 | 1 | 1 | 1 | 1 | 10 |
| Waugh et al., 2016 | 1 | 0.5 | 1 | 1 | 1 | 1 | 1 | 1 | 1 | 1 | 9.5 |
| Burciu et al., 2017 | 1 | 0.5 | 0.5 | 1 | 1 | 1 | 1 | 1 | 1 | 0.5 | 8.5 |
| Kirke et al., 2017 | 1 | 1 | 1 | 1 | 1 | 1 | 1 | 1 | 1 | 0.5 | 9.5 |
| Mantel et al., 2018 | 1 | 1 | 1 | 1 | 1 | 1 | 1 | 1 | 1 | 1 | 10 |
| Bianchi et al., 2019 | 1 | 1 | 1 | 1 | 1 | 1 | 1 | 1 | 1 | 0.5 | 9.5 |
| Chirumamilla et al., 2019 | 1 | 0.5 | 0.5 | 1 | 1 | 1 | 1 | 1 | 1 | 1 | 9 |
| Cracien et al., 2019 | 1 | 0.5 | 0.5 | 1 | 1 | 1 | 1 | 1 | 1 | 1 | 9 |
| Mantel et al., 2019 | 1 | 0.5 | 1 | 1 | 1 | 1 | 0.5 | 1 | 1 | 1 | 9 |
| Liu et al., 2020 | 1 | 1 | 1 | 1 | 1 | 1 | 1 | 1 | 1 | 1 | 10 |
| Tomic et al., 2021 | 1 | 1 | 1 | 1 | 1 | 1 | 1 | 1 | 1 | 1 | 10 |
| Wu et al., 2022 | 1 | 1 | 1 | 1 | 1 | 1 | 1 | 1 | 1 | 1 | 10 |
| Yang et al., 2024 | 1 | 1 | 1 | 1 | 1 | 1 | 1 | 1 | 1 | 1 | 10 |
